# 蝮蛇咬伤致腹腔出血1例报告并文献复习

**DOI:** 10.3760/cma.j.cn121090-20241228-00601

**Published:** 2024-12

**Authors:** 睿知 李, 晋 李, 文辉 赵, 晖 徐, 丹 赵

**Affiliations:** 江西中医药大学附属医院重症医学科，南昌 330000 Department of Critical Care Medicine, Affiliated Hospital of Jiangxi University of Traditional Chinese Medicine Jiaotong University, Nanchang 330000, China

## Abstract

毒蛇咬伤是一种急性全身性的中毒性疾病，主要可表现为弛缓性瘫痪、全身溶肌、凝血障碍、肾功能损害、心脏毒性、咬合节段局部组织损伤。本文报道1例少见的蝮蛇咬伤第5天致自发性腹腔出血的患者，予以追加抗血清注射液、加强纠正凝血功能后病情好转，最终腹腔积血吸收。

## Abstract (en)

Venomous snake bite is an acute systemic toxic disease, mainly manifested as: flaccid paralysis, systemic muscle lysis, coagulation disorders, bleeding, renal dysfunction, cardiac toxicity, and local tissue damage to bite segments. This article reports a rare case of spontaneous abdominal bleeding caused by a pit viper bite on the 5th day. After receiving additional anti serum injection and strengthening the correction of coagulation function, the patient's condition improved and eventually the abdominal bleeding was absorbed.

蝮蛇毒是以血液毒为主兼有神经毒素的混合毒，被蝮蛇咬伤后病情可迅速进展，同时可伴有心脏、肝脏、肾脏损伤，以至并发多脏器功能衰竭[1]–[2]。本文报道1例蝮蛇咬伤致自发性腹腔出血患者的诊疗过程，结合该病例分享治疗体会，为此类疾病的诊疗提供参考。

## 病例资料

患者，女，75岁，汉族。因“蝮蛇咬伤致右踝部肿痛38 h余”于2024年5月20日入院。患者38 h前被蝮蛇咬伤右踝，于当地诊所外敷草药（具体不详），症状未缓解，出现右踝肿胀加重伴口渴、尿少、乏力等症状。既往左肾萎缩病史（具体原因不明），否认毒蛇咬伤后外伤史。体格检查：体温36.5 °C，脉率120次/min，呼吸32次/min，血压138/76 mmHg。神志清楚，急性病容，呼吸急促，双肺可闻及细湿啰音，右下肢肿胀伴皮肤青紫瘀斑（图1），心脏及腹部查体未见明显异常。血常规：WBC 22.31×10^9^/L，中性粒细胞占93.3％，HGB 124 g/L，PLT 43×10^9^/L。血生化：ALT 193.2 U/L，AST 761.3 U/L，血肌酐214.5 µmol/L，肌酸激酶34 269.1 U/L。PT系列：D-二聚体1.29 mg/L，凝血酶原时间10 s，国际化标准化比值（INR）0.99，纤维蛋白原4.54 g/L，凝血酶时间14 s，活化部分凝血活酶时间26.4 s，纤维蛋白（原）降解产物4.5 mg/L。入院诊断：毒蛇咬伤（蝮蛇，右踝，重度）。入院后予抗蝮蛇血清12 000 IU，水化碱化，抗感染，预防破伤风，创面中药涂擦治疗。入院第2天转入ICU，入ICU时生命体征：体温36.8 °C，脉率123次/min，呼吸35次/min，血压148/95 mmHg（硝酸甘油0.33 µg·kg^−1^·min^−1^），血气分析：pH 7.34，PO_2_ 86 mmHg,PaCO_2_ 32.7 mmHg，HCO_3_ 17.9 mol/L，BE −7 mmol/L，乳酸1.0 mmol/L。血常规：WBC 16.71×10^9^/L，HGB 91 g/L，PLT 54×10^9^/L。血生化：ALT 336.9 U/L1，AST 971.6 U/L,肌酐327.0 µmol/L，肌酸激酶44092 U/L。PT系列：D-二聚体1.78 mg/L，凝血酶原时间10 s，纤维蛋白原3.49 g/L，凝血酶时间16 s，活化部分凝血活酶时间23 s，纤维蛋白（原）降解产物2.81 mg/L。2024年5月21日胸腹部CT平扫示：两下肺慢性炎性改变，左肾萎缩，右肾周围可见渗出。ICU予经鼻高流量氧疗、血液净化（HP+CVVH）、镇静镇痛、纠正贫血、抗感染治疗。入院第4天患者出现休克伴血红蛋白、血小板进行性下降，床旁腹部彩超示腹腔积液，复查胸腹部CT示：肝、脾脏周围积液，脾脏包膜欠清晰，脾胃间隙系膜明显增粗（图2）。予诊断性腹穿抽出不凝血液，遂立即予输同型去白细胞悬浮红细胞、补液扩容，中医外科会诊后追加抗五步蛇血清注射液4000 IU，补充人纤维蛋白原1 g。经治疗患者血红蛋白未进行性下降，肾功能改善，但因经济原因患者入院第6天自动出院。出院后第46天对患者回访右下肢肿胀消退（图3），尿量1500 ml/d，临时血液透析管已拔除，血常规：HGB 99 g/L，PLT 165×10^9^/L，凝血功能检测均正常，全腹部CT示腹腔积血完全吸收（图4）。

**图1:**
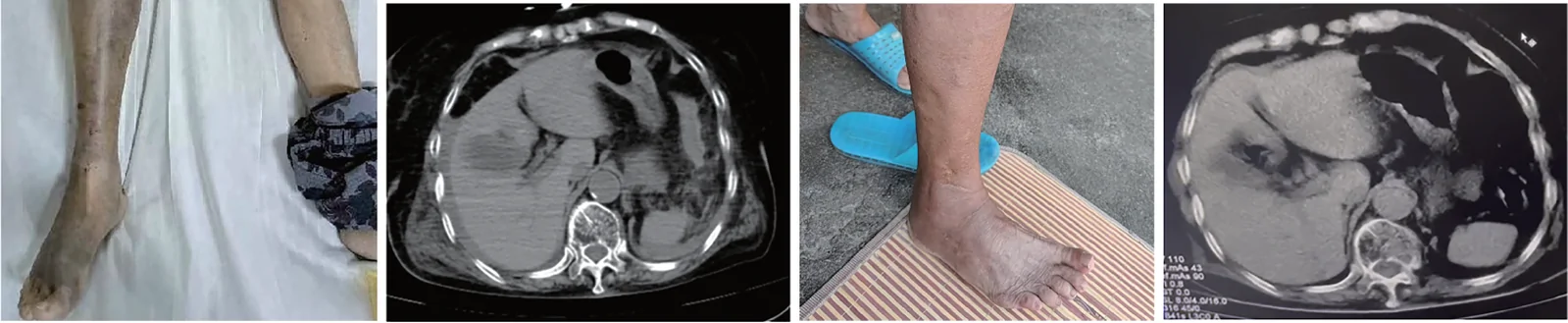
患者入院时右下肢 **图2** 患者入院时腹部CT提示腹腔积液，脾脏包膜欠清晰 **图3** 患者出院后第46天回访时右下肢 **图4** 患者出院后第46天腹部CT提示腹腔积血吸收

## 讨论及文献复习

据WHO2022年统计全球有超过58亿人面临蛇咬伤毒害的风险[3]，据不完全统计我国每年发生25～28万例毒蛇咬伤[4]。蛇毒是自然界成分复杂的天然高效毒素混合物，主要由蛋白（占蛇毒干重的90％～95％）和非蛋白成分组成。毒蛇种类繁多，每种毒蛇可同时含多种不同毒素。蛇毒蛋白因作用位点不同可产生多种毒性效应，其对血液系统损害主要原因有：①蛇毒Ⅴ因子活化剂、Ⅹ因子活化剂、凝血酶原激活剂和凝血酶样酶等产生促凝效应；②Ⅸ/Ⅹ结合蛋白、蛋白C激活剂、凝血酶抑制剂和PLA2等表现为抗凝特性；③纤维蛋白溶解酶和纤溶酶原激活剂等具纤维蛋白溶解活性；④蛇毒金属蛋白酶、去整合素和C型凝集素会直接破坏血管壁[5];⑤抑制血小板活性从而降低其止血的有效性；病理性地促进血小板参与止血[6]。毒液诱发的消耗性凝血病（VICC）是由促凝毒素激活的凝血途径，导致凝血因子消耗和凝血障碍。VICC所致的出血轻重程度不一，可以从轻微的咬伤部位或穿刺点的渗血，到严重的甚至危及生命的出血，如自发性的颅内出血（该情况通常伴高血压），或是在创伤后发生的头、胸腔或者其他脏器出血。

本病例为蝮蛇咬伤所致自发性腹腔出血,国内外均较少见。回顾国内外蛇咬伤致腹腔出血的病例报道，其致腹腔出血病因呈多样性。Iwakiri等[7]和Kim等[8]分别报道了1例毒蛇咬伤致缺血性结肠炎病例，Ahn等[9]报道了1例毒蛇咬伤致肝坏死和破裂所致的非创伤性腹腔出血，脾破裂病例亦偶有报道。脾脏是一个富含血管的脆弱器官，虽多数脾破裂是由外伤引起的，或继发于肿瘤、感染和侵袭性疾病，然而在凝血功能受损的患者中可发生自发性脾破裂。本病例腹部CT见脾脏包膜欠清晰、脾胃间隙系膜明显增粗，遂笔者认为该患者脾脏出血可能性大。大部分已被报道的蛇咬伤致自发性脾破裂的病例进行了介入和（或）手术切除脾脏治疗[10]–[13]，本病例因缺乏明确证据支持患者存在腹腔血管活动性出血，考虑VICC所致凝血功能障碍，遂监测生命体征尚稳定的情况下采取追加抗五步蛇毒血清4000 IU治疗。因毒蛇种类繁多，目前尚缺乏针对单独每一种毒蛇的抗蛇毒血清，我国现有4种马免疫单特异性F（ab）2型抗蛇毒血清，分别是抗眼镜蛇毒血清、抗银环蛇毒血清、抗蝮蛇毒血清以及抗五步蛇毒血清。入院时该患者已使用抗蝮蛇毒血清，但仍然发生了自发性出血事件，血液毒为当前突出问题，五步蛇毒素大量消耗凝血因子为主，遂基于异种血清联合的治疗原则追加了抗五步蛇毒血清，而不是抗蝮蛇毒血清。Kang等[12]报道1例患者被毒蛇咬伤2 h后使用抗蝮蛇毒血清1支，即使病程第3天每6 h追加2支抗蝮蛇毒血清，第4天患者仍然出现了脾假性动脉瘤和脾周血肿伴大腹腔积血。这可能与VICC对抗蛇毒血清的反应存在明显的差异有关。在非洲进行的一项为期12年的前瞻性研究报道，纳入60例患者比较了凝血时间恢复的时间，47例注射了抗蛇毒血清，13例未注射，未治疗组恢复到纤维蛋白原水平超过1 g/L的时间为7.5 d，而抗蛇毒血清组为40 h，差异具有统计学意义（*P*<0.0001）[14]；而在一项澳大利亚研究中，比较了咬伤后6 h内给予抗蛇毒血清的患者和咬伤后6 h以上给予抗蛇毒血清的患者的PT/INR恢复情况，两组之间的PT/INR改善差异无统计学意义[15]。

VICC的治疗主要是通过中和促凝毒素并使凝血因子恢复以预防大出血及由此产生的并发症，需要注意的是抗蛇毒血清不能修复由凝血功能障碍引起的损伤，如关键的器官损伤，也不能“关闭”在凝血功能障碍期间激活的继发性现象。因此，一旦检测到凝血功能障碍，应尽早给予正确的抗蛇毒血清；对于已存在活动性出血的患者，可以使用凝血替代疗法加速凝血功能的恢复[16]。抗蛇毒血清是VICC的主要治疗方法，但仍缺乏高质量的证据支持其有效性；有证据支持在VICC出血患者中使用新鲜冰冻血浆；没有证据支持肝素的使用[17]。

毒蛇咬伤后出现凝血障碍，必须尽早发现患者任何危及生命的出血，如果观察到灾难性出血，应给予额外的抗蛇毒血清。此外，如果大出血无法得到控制，应考虑早期血管介入或手术。
